# Seeing through collagen: integrative pro-regenerative corneal implants for clearer future

**DOI:** 10.1038/s41536-026-00471-0

**Published:** 2026-03-14

**Authors:** Xia Huang, Mohammad M. Islam, Stephanie L. Watson, Hirak K. Patra

**Affiliations:** 1 College of Chemistry, Sichuan University, Chengdu, China; 2 Department of Surgical Biotechnology, Division of Surgery and Interventional Science, University College London, London, UK; 3 Department of Ophthalmology and Visual Sciences, University of New Mexico School of Medicine, Albuquerque, NM USA; 4 Sydney Medical School, The University of Sydney, Sydney, NSW Australia

**Keywords:** Biological techniques, Biotechnology, Diseases, Engineering, Materials science, Medical research

## Abstract

Collagen-based artificial corneas are a promising regenerative alternative to scarce donor tissue. However, their clinical success is limited by mechanical weakness, enzymatic degradation, and poor bio-integration. This review provides a comprehensive analysis of the current state and the future strategies to optimize collagen for making pro-regenerative corneal implants, enhancing the performance, functionality, and clinical viability of the collagen-based artificial cornea as a substitute for donor tissue.

## Introduction

The cornea is the outermost, transparent, and avascular tissue of the eye, which functions as a structural barrier to protect the eye from infection, infiltration by foreign bodies from outside, and ultraviolet radiation. For optimal vision, the cornea provides two-thirds of the eye’s ability to focus light and must stay perfectly clear^1,2^. The cornea is one of the most sensitive tissues in the body due to its rich innervation. Corneal blindness is a significant contributor to vision loss globally and stems from a variety of conditions, such as infection, trauma, autoimmune disease, vitamin A deficiency, dystrophy, and ectasia following trauma^3^. Corneal blindness affects approximately 10 million individuals worldwide and is prevalent in low-income countries within the developing world, where there is a scarcity of resources and infrastructure^4–7^. Corneal transplantation (i.e., keratoplasty) is the most common transplantation procedure performed clinically and a leading option for the treatment of corneal blindness. The shortage of donor corneal tissues restricts the number of corneal transplants that can be performed each year^8,9^. Artificial corneas can serve as an alternative to donor corneal tissue, potentially enabling the global treatment of corneal blindness.

The native cornea consists of five distinguishable layers: three cellular layers (epithelium, stroma, and endothelium) and two acellular layers (Bowman’s and Descemet’s membranes)^10,11^. In 2013, a sixth corneal layer was proposed between the corneal stroma and Descemet’s membrane^12^. Among these layers, the corneal stroma is the thickest layer, accounting for 80–90% of the cornea’s approximate 500 µm thickness. The corneal stroma is primarily composed of collagen fibrils, proteoglycans, and keratocyte cells. Normally, keratocytes in the corneal stroma remain inactive, but they can become active in response to injury or disease, aiding in wound healing and synthesizing new extracellular matrix^13^. In response to disease or injury, keratocytes can undergo fibroblastic transformation, which leads to the production of an unorganized extracellular matrix component followed by dense scar formation, disrupting proper cornea hydration and leading to a loss of transparency. In cases where the scarring is deep, penetrating keratoplasty is necessary to restore vision. Materials for artificial cornea should ideally be transparent, or become transparent once implanted into the cornea, and have sufficient strength for suturing or gluing into place, and to withstand trauma while providing the necessary flexibility and adaptability required by patients^14^. The success of a tissue-engineered artificial cornea hinges on its material composition, which governs the host response. Artificial corneas are laboratory-made constructs, typically consisting of synthetic materials with or without the addition of biological components, for the substitution or to complement the native human cornea to replace the need for donor tissue for vision restoration. An optimal corneal replacement must exhibit transparency, non-cytotoxicity, genetic stability, mechanical robustness, and seamless integration with ocular tissues, while maintaining nutrient permeability and facilitating epithelial regeneration. Critically, it should also resist protein adsorption to avoid opacification and preserve long-term visual acuity^14,15^. The transmittance of native corneal tissue ranged from 80% to 94% across wavelengths of 450–600 nm and 95% to 98% in the 600–1000 nm range. Light transmission and corresponding transparency are fundamental criteria for a functional cornea^16,17^. Artificial corneas offer a solution to religious, cultural, financial, and regulatory challenges associated with donor corneal tissue, and they can restore significant vision in severe cases of corneal blindness where donor tissue is ineffective. Artificial corneas can be engineered to reduce the risks of immune rejection, graft failure, and complications from ocular surface diseases^18^.

The Boston Keratoprosthesis (BKpro) is one of the most widely used artificial corneas and was first introduced in the 1970s^19^. It is a collar button design, consisting of a front plate which is made of medical-grade polymethylmethacrylate (PMMA) with an optical stem, a corneal allograft button, and a back plate. The primary reason for BKPro use is to treat advanced-stage corneal diseases, such as in patients with multiple failed donor transplants. However, BKPro requires donor corneal tissue as a carrier for the prosthetic, contributing to issues of scarcity and cost. Beyond tissue sourcing, challenges include managing potential device retention complications and optimizing the final visual results. Moreover, risks intrinsically linked to the donor graft component, such as failure, immune reactions, and disease transmission, remain ongoing concerns with BKpro grafts^20^. The AlphaCor^TM^, a fully synthetic artificial cornea, was developed to address some of the limitations of human donor tissue in high-risk cases where traditional penetrating keratoplasty is contraindicated due to a high likelihood of graft rejection or failure. Though it can restore functional vision in some patients, unfortunately, achieving “full visual potential” is uncommon due to persistent challenges. The use of the AlphaCor^TM^ is associated with complications such as corneal stromal melting (linked to material-tissue interactions), postoperative ocular herpes simplex virus reactivation, chronic inflammation, and optic surface deposition or degradation (requiring device replacement or adjunct therapies), which have limited its clinical adoption^21^. Despite advancements in bioengineered corneal substitutes, current artificial corneas remain inferior to donor corneal transplants in terms of long-term biocompatibility, optical clarity, and complication rates, restricting their use to niche scenarios where donor tissue is unviable^18,22^.

Collagen emerges as the optimal choice as a biomaterial, constituting 71% of the corneal extracellular matrix (ECM) and serving as the primary structural scaffold for corneal transparency and mechanical stability. Its hierarchical fibrillar organization is essential for maintaining corneal morphology and optical clarity. Beyond its natural prevalence in corneal tissue, collagen offers unparalleled advantages for tissue engineering: it is abundant, cost-effective, and easily sourced from various biological tissues, while its well-established processing methods facilitate scalable production^23^. Collagen could therefore be considered as the superior ‘building block’ biomaterial for artificial cornea fabrication. Collagen provides an inherently bioactive matrix that promotes cell adhesion, proliferation, and ECM remodeling, key requirements for corneal regeneration. Its low immunogenicity and ability to mimic embryonic developmental cues make it uniquely suited for recapitulating organogenesis in an artificial cornea. Given these properties, collagen stands as the most physiologically relevant and functionally superior for bioengineered corneas, bridging the gap between synthetic prosthetics and natural tissue regeneration^24^. However, collagen’s ability to form intra- and interfibrillar organization to form hydrogels is inherently shown to have low mechanical strength without covalent crosslinking, which expresses a significant limitation for their use as artificial corneal implants^25^. Their enzymatic susceptibility further restricts their utility in severely diseased or inflamed eyes, where proteolytic degradation may compromise the implant stability. To overcome these challenges, several strategies have been developed to enhance mechanical robustness through physical and chemical modifications. The functional amine and carboxyl groups on collagen molecules can be exploited to crosslink, tailoring its chemical and physical properties through chemical modifications^26^. Natural collagen complexes, formed by combining different molecular collagen types, can inspire biomimetic designs. By replicating these hierarchical structures in synthetic collagen-based materials, it may be possible to achieve mechanical properties comparable to native corneal tissue, improving resistance to internal and external stresses^27^.

We provide a critical and comprehensive overview of emerging techniques for engineering collagen hydrogels to create viable substitutes for artificial corneas. We examine collagen modifications, advancements in fabrication techniques, crosslinking strategies, and functionalization methods, all guided by a rational design approach. Despite the progress in this field, universally accepted strategies for developing collagen-based artificial corneas remain far from the clinic. Given the crucial role of collagen in the development of artificial corneas, this review will focus on the parameters optimizing the performance of these materials. This article is intended to evaluate current strategies to identify the knowledge gap and guide potential future research in integrating collagen into artificial corneal implants.

## Collagen properties

**Fig. 1:**
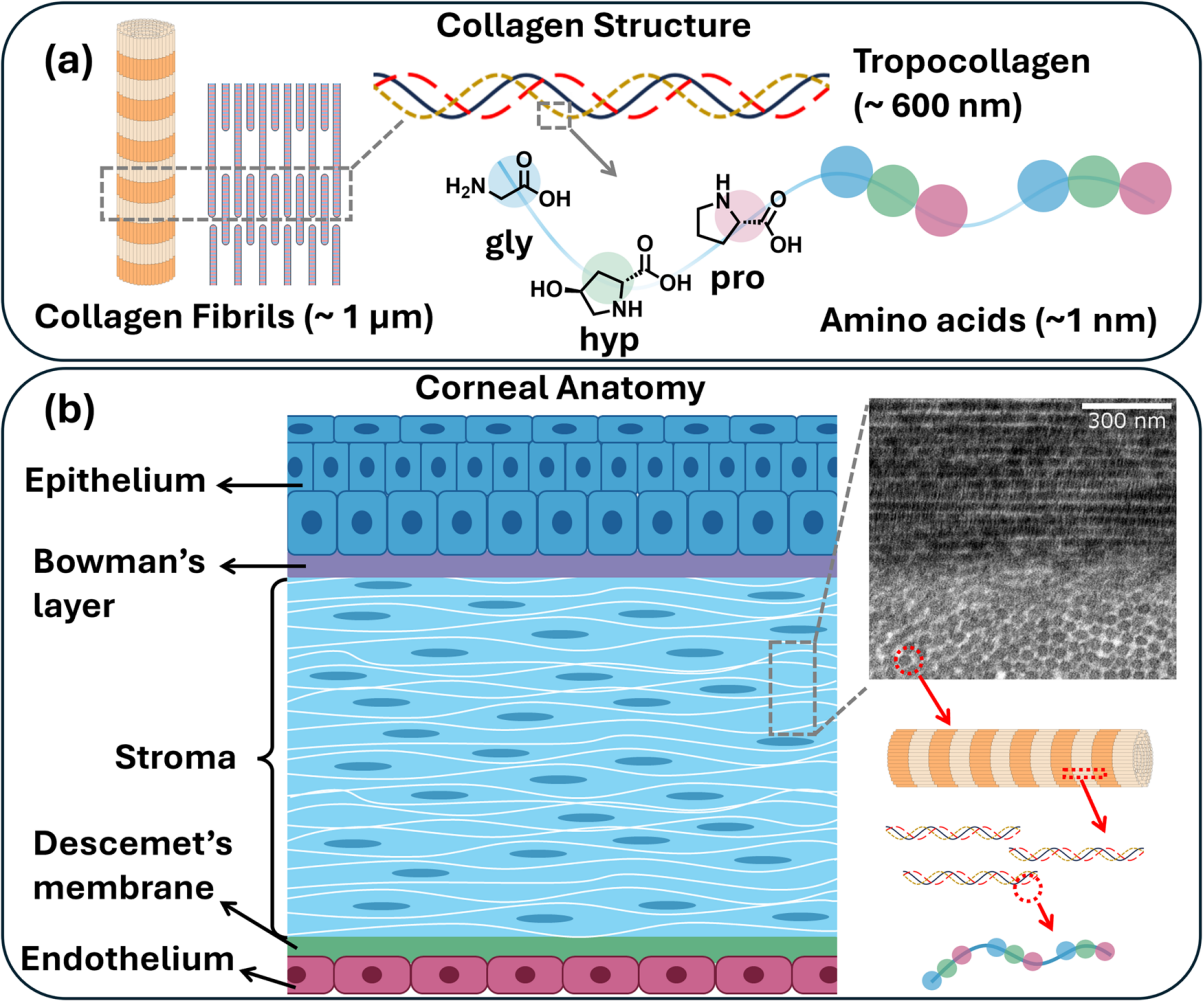
Native corneal collagen arrangement. **a** Collagen fibrils and triple-helical Structure and **b** Hierarchical arrangement of collagen in the cornea, reproduced from^41^.

Collagen is an abundant fibrous protein that forms most of the ECM in all animals^28,29^. The word collagen derives from Geek word ‘Kolla’, signifying ‘glue’, and as biological polymer. It is the main component of connective tissues, making up over 30% of the total protein content in the animal body^30^. As a biomaterial, the first application of collagen can be traced back to 1881, when surgeons Joseph Lister and William Macewen developed collagen-based sutures from sheep intestine^31,32^. By the year 1993, the U.S. Food and Drug Administration (FDA) had approved the first collagen-based bone implants^33^. This marked the beginning of a new era in the application of collagen in the field of biomedicine. Numerous innovations in collagen manipulation have extended the collagen application in tissue engineering^34^. Collagen has a helix hierarchical structure composed of three associated alpha-helix polypeptide chains linked through hydrogen bonds between hydroxylysine and l-hydroxyproline and by covalent bonds, forming a highly organized 3D structure (Fig. 1a). Tropocollagen is the building unit of collagen with a length of 300 nm and a diameter of 1.6 nm^35^. Each polypeptide chain of collagen is a repetitive (Gly-Xaa-Yaa)_n_ tri-amino acid unit, where glycine is every third residue^29^. Xaa and Yaa can be any amino acid, and the positions are often occupied by proline (Pro) and hydroxyproline (Hyp), as identified by Fischer, making Pro-Hyp-Gly the most prevalent sequence within collagens^36,37^. Till now, 29 different types of collagens have been identified. Each type varies in the shape of the globular domain, interval in the triple-helix structure, and the length of the collagen. However, only a few of them are used in exploring collagen-based biomaterials. Type I collagen, the predominant collagen in animal tissues, is the most extensively utilized collagen in biomaterial applications due to its availability from both animal-derived natural sources, synthetic, and biosynthetic sources. Natural Type I collagen is primarily derived from animal tissue sources, including bovine, porcine, rat, and human tissues, as well as marine organisms. Alternatives, such as recombinant collagen, have been engineered to circumvent immunogenic risks associated with natural sources while ensuring batch-to-batch consistency^34^. Recombinant strategy enables the production of high-purity, pathogen-free collagen through heterologous expression systems, including mammalian cells, insect cells, yeast cultures, and plant-based platforms^38^. The high cost of production, low yield, and the limited protein expression are considered to hinder the scalability of recombinant technologies^39^. Consequently, animal-sourced collagen remains the gold standard in both research and clinical settings, balancing cost-effectiveness with functional reliability^40^.

The corneal stroma, is a collagen-rich extracellular matrix in which Type I collagen predominates, forming 80–90% of stromal fibrils. While other minor collagens (e.g., types V, VI, and XII) regulate fibril diameter, spacing, and matrix interactions^23,41,42^. These fibrils are composed of collagen molecules arranged in a staggered, quarter-staggered array, producing a periodic banding pattern (D-periodicity) of 65 nm as shown in Fig. 1b. Human stromal fibrils exhibit remarkable uniformity, with diameters tightly controlled at 32.5 ± 1.5 nm, a critical determinant of corneal transparency^16,23,43^. Essential for corneal transparency and strength, a lattice-like architecture is formed within the stroma by roughly 200 to 250 lamellae of collagen fibrils organized orthogonally to one another (Fig. 1b)^41^. This orthogonal alignment minimizes light scattering by offsetting refractive index variations between adjacent lamellae, ensuring optical clarity while conferring biomechanical resilience^44,45^. This meticulous collagen organization and architecture contribute to the cornea’s ability to withstand external forces while maintaining its structural integrity. Anterior lamellae interweave and anchor into Bowman’s membrane, a specialized acellular layer that interfaces with the epithelium and enhances anterior stromal rigidity^46,47^. This rigidity, combined with the lamellar lattice, maintains corneal curvature that is essential for refracting light onto the lens and retina. Therefore, the intricate architecture of the corneal stroma exemplifies a harmonious balance between transparency and mechanical strength, essential for maintaining optimal vision^48^. Along the corneal collagen fibril major axis, molecules are arranged with the usual collagen quarter stagger, and the collagen molecules are stabilized by intermolecular covalent crosslinks, which contribute significantly to tissue integrity and provide mechanical stability of cornea^49,50^. However, extracted collagen often differ from its native form for the use of corneal substitutes. It typically lacks organized and controlled assembly, covalent crosslinking, and integration of diverse molecular forms found in native collagen complexes. These factors result in insufficient mechanical strength to withstand internal and external stresses. If the native collagen complexes can be mimicked in artificial collagen-based biomaterials, it is hoped that similar mechanical properties to natural collagen can be maintained, and covalent crosslinks between collagen molecules can further stabilize the fibrillar network, enhancing tensile strength and resistance to deformation for clinical success^27^. Therefore, enhancing the structural biomimicry and stability of collagen-based constructs is paramount for corneal regenerative applications.

The collagen-based biomaterials employed for engineering corneal substitutes must replicate the structural and functional requirements of the native cornea. The overview of physical properties of collagen-based artificial cornea in comparison with native cornea is summarized in Table 1. Collagen processing technologies have enabled, in different ways, the manufacture of structurally versatile materials with varying properties with regard to shape, mechanics, physiological behavior, and handling. These technologies aim to enable the collagen-based material to be manufactured with suitable mechanical toughness, biocompatibility, transparency, appropriate biodegradability, along with clinical compliance via intensive chemical, mechanical, and physical treatment, or a combination process^51^. Chemical methods involve chemical agents that interact with collagen through amino- or carboxyl- functional groups, resulting in the formation of crosslinks between specific collagen molecules. Physical methods, on the other hand, encompass techniques such as heating, drying, and irradiation^52^. The manipulation and configuration of collagen-based biomaterials are pivotal for creating innovative structures suited for clinical corneal applications. This strategic manipulation not only enhances the biomaterials’ functionality but also broadens their applicability in ophthalmological treatments, thereby contributing to advancements in corneal therapy and vision restoration. The following section discusses the processing of collagen into an artificial cornea.

**Table 1:** Overview of the physical characteristics of recent collagen-based artificial corneas

Materials	Crosslinking	Fabrication and form	Key Mechanical Property			Transmittance	Clinical status	Collagenase resistance	Ref.
			Elastic modulus (MPa)	Ultimate strength (MPa)	Suture retention				
Human cornea	-	-	3–13 MPa^176^	3.81 ± 0.04 Mpa^177^	0.35 N^66,116,178^	Refractive index 1.36-1.37^143,179^	-	-	^66,116,143,176–179^
Cyclodextrin Modulated Collagen	No crosslinking	Self-assembly regulation	2.4 MPa	2 MPa	0.29 N	77% at 550 nm	In vivo	-	^59^
Collagen	1,4-Butanediol diglycidyl ether	Molding	2.86 MPa	0.21 MPa	-	Refractive index: 1.35 86%	In vitro	12 h	^99^
Collagen	Glutaraldehyde	Electrical Signal Initiates Kinetic Assembly	-	5.22 ± 0.73 MPa	1.75 ± 0.21 N	80%	In vivo	40 h No weight loss	^64^
Glycerol Modulated Collagen	Hydroxylmethyl bicyclic oxazolidine crosslinking	Self-assembly regulation Molding	1.975 MPa	1.6 MPa	0.49 N	90%	In vivo	10% weight loss in 70 h	^63^
Collagen and pyrene conjugated dipeptide amphiphile	Hydrophobic interactions between pyrene	No chemical crosslinking	-	-	0.49 ± 0.09 N Suturable	80%	In vitro	-	^122^
Recombinant human collagen	EDC/NHS	Molding	22 MPa	2.2 MPa	Suturable	Refractive index : 1.35 78.2 ± 0.3 ~ 92.6 ± 0.9	In vivo	-	^90^
Collagen	Multi-functional polyethylene glycol (PEG)-N-hydroxysuccinimide (NHS)	In situ filler	Storage modulus: 1.32 MPa	-	-	80%	-	12 h	^108^
Collagen	N-Cyclohexyl-N′-(2-morpholinoethyl) carbodiimide metho-p-toluenesulfonate (CMC)/NHS	Molding	10.40 ± 2.28 MPa	1.099 ± 0.980 MPa	Suturable	Refractive index : 1.34 88.06 ± 1.94%	In vivo	28 h	^91^
collagen–gelatin	EDC/NHS	Molding	6.28 ± 1.17 KPa	16 KPa	-	80.43%	In vitro	7 days Fully degradation	^180^
Collagen–phosphorylcholine	EDC/NHS	interpenetrating network Molding	2.09 ± 1.12 MPa	0.69 ± 0.17 Mpa	Suturable	Refractive index : 1.35 87.9 ± 2.2%	In vivo	-	^120^
Collagen TEMPO oxidized nanocellulose	EDCM/NHS and riboflavin (Vitamin B2)	TEMPO oxidized nanocellulose linked with EDC/NHS-crosslinked collagen Molding	10.81 GPa	797.9 kPa	Suturable	85%	In vivo	8 h 50% mass loss	^149^
Methacrylic anhydride (MA) modified recombinant human collagen	Lithium phenyl-2,4,6-trimethylbenzoylphosphinate (LAP) UV crosslinking	Photolithography mask molding	34.3 5.2 kPa	Compressive stress 40 ~ 120 KPa	Suturable	90%	In vitro	-	^139^
Collagen and Hyaluronic Acid	SPAAC reaction and Thiol-ene reaction Thiolated HA (HA-SH) Methacrylated HA (HA-MA) Azido-modified Col (Col-azide) DBCO-modified Col (Col-DBCO)	Gel filler Defects filling	Storage modulus: 1 MPa	-	Suture-less filler	Refractive index :1.341 94%	In vivo	-	^118^
Hyaluronate-collagen	SPAAC reaction HA-azide Col-DBCO	Gel filler Defects filling	-	-	-	Refractive index : 1.34 95%	In vivo	-	^153^
PEG-stabilized collagen–chitosan	Hybrid polymer network EDC/NHS poly(ethylene glycol) dibutyraldehyde	Molding	1508.0 ± 59.0 KPa	220 KPa	Suturable	90%	In vivo	Non in vivo degradation for 120 days	^181^
Gelatin methacrylate and collagen	GelMA: I2959 UV crosslinking Collagen: EDC/NHS	3D printing	55 KPa	140 Kpa	Suturable	65 ~ 80% at 550 nm	In vivo	-	^182^

### Physical interactions

**Fig. 2:**
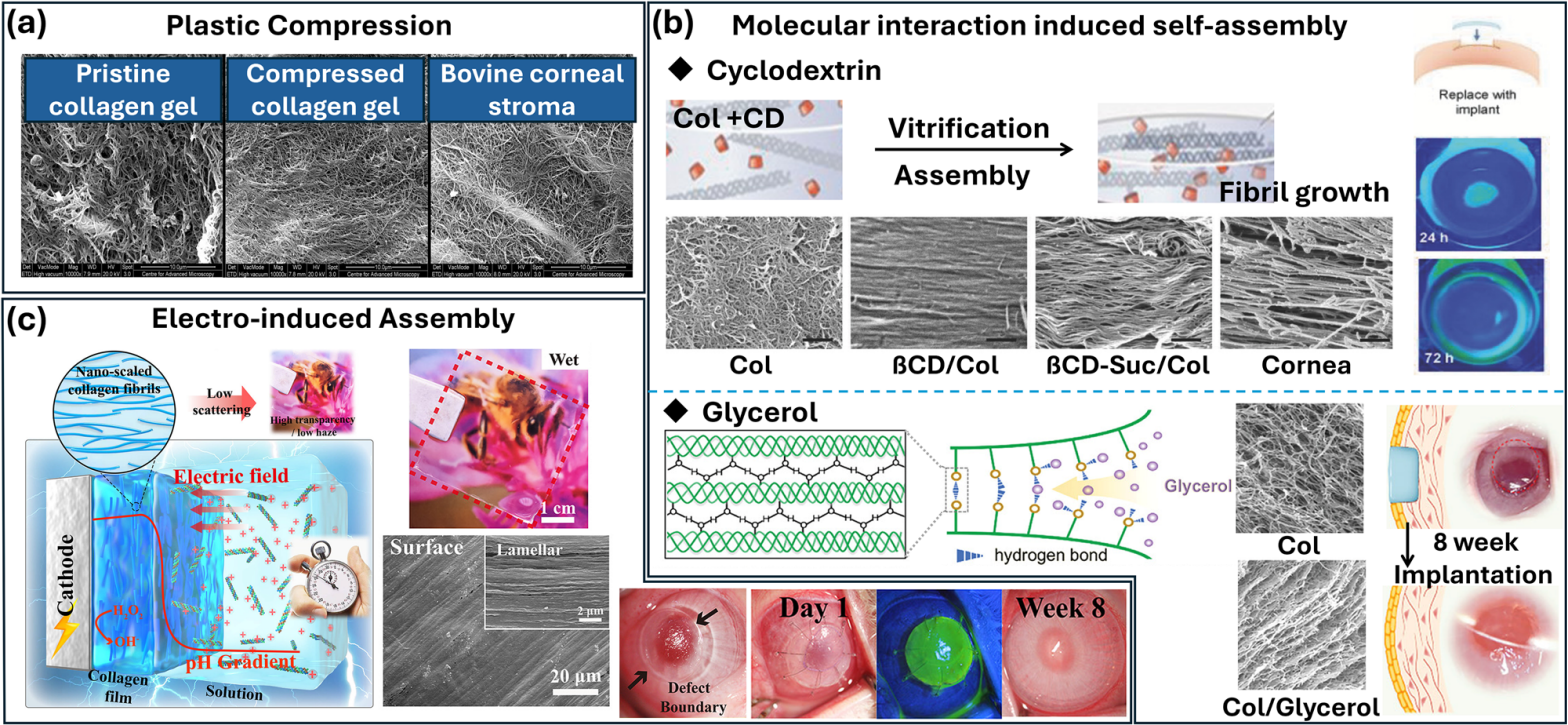
Physical interaction strategies for the preparation of collagen hydrogels for corneal substitutes. **a** Compression, **b** Cyclodextrin and glycerol molecule assisted self-assembly^59,63^, and **c** Electrical assisted self-assembly and the corresponding photographs before and after transplantation^64^.

Physical interactions play a crucial role in the preparation and optimization of collagen hydrogels for use as cornea substitutes. Collagen is a fibrillar and easy to tear without modification. The matrix microstructural anisotropy and alignment can also affect, to a great degree, its mechanical property, optical property, and cellular organization^53^. Plastic compression strategy is a chemical-free, biocompatible approach invented 20 years ago. In this strategy, collagen hydrogels are sandwiched between layers of nylon and blotting papers and are then subjected to unconfined compression. Compressed collagen shows great promise as a scaffold for corneal stroma analogs. It effectively incorporates keratocytes and mimics the mechanical and structural environment of the native cornea with its dense collagen fibrillar structures (Fig. 2a)^54,55^. By controlling the propensity of collagen to form an organized matrix upon dehydration, the collagen spontaneously becomes dense, homogeneous, and organized in structure, with increased stiffness consistent with improved mechanical strength. However, the plastic-compressed collagen membrane has poor optical transmittance and permeability. And this extreme dehydration reduces collagen fibril plasticity, making the material prone to cracking during handling^56–58^. In an effort to form a lamellae arrangement of collagen fibrils without sacrificing the material permeability, cyclodextrin has been used to modulate collagen fibrogenesis and arrangement. Using cyclodextrin, highly transparent and mechanically robust collagen vitrigel implants can be created with a unique lamellar-arranged ultrastructure that mimics the native cornea. Cyclodextrin interacts with collagen molecules during early gelation in collagen hydrogels and promotes vitrification processes through controlled dehydration under defined temperature and humidity conditions, thereby directing fibril assembly into lamellar stacks (Fig. 2b). The cyclodextrin/collagen network is mechanically robust with good transparency and demonstrates the ability for tissue integration and re-epithelialization, which demonstrates their potential as a biomimetic corneal substitute. The collagen fiber diameter, orientation, and lamella development have been found to depend on the size and chemical functionality of the cyclodextrin combined with the gels^59^. Although certain progress has been made in the fabrication of cyclodextrin-based collagen hydrogels and their applications as corneal repair material, further efforts and animal experiments are still required to translate clinically, and their mechanism should be taken seriously to be re-evaluated in humans due to the more complex systems^60–62^. Additionally, glycerol, a strong polar green solvent, serves as a regulator in disrupting and reorganizing hydrogen bonds within thermally assembled crude collagen fibrils. By enhancing hydrogen bonding, glycerol weakens intermolecular crystallization at the molecular level, facilitating collagen chain aggregation and controlled spacing at the nanoscale (Fig. 2b). This leads to the formation of small, ordered microfibrillar clusters at the microscale, ultimately reorganizing collagen into a lamellar structure with optimized fibril diameter and spacing. This structural adjustment balances collagen thickness, transparency, and permeability, making glycerol treatment a simple yet effective method for tailoring collagen hydrogel to create bionic artificial corneal substitutes^63^. The network was further stabilized by oxazolidine to form a collagen-based cornea implant, which can effectively support epithelialization and stromal remodeling, and restore a transparent cornea within 8 weeks of corneal transplantation. However, as a humectant, glycerol may acquire excessive water molecules, resulting in swelling and altering the hydrogel shape and transparency. Additionally, the risk of glycerol leaching may lead to structural instability and interfere with cell adhesion and cell function.

In order to further control the complex three-dimensional geometries and customize collagen-based artificial corneas with specific curvatures, Lei et al. proposed electrical signals to induce the kinetic assembly of collagen^64^. Under the applied electric field, the positively charged collagen triple-helix molecules migrate rapidly and accumulate near the cathode area, triggering assembly of the collagen (Fig. 2c). The further pH change induced by the cathodic reaction drives collagen assembly into microfibrils at regions where the pH approaches its isoelectric point 4.5, with intermolecular interactions leading to the formation of a crosslinked collagen hydrogel film. The compacted lamellar organization of collagen fibrils can increase transparency by decreasing light scattering, and such ultrastructure was further chemically crosslinked to offer clinically required physicochemical properties for artificial cornea applications. Physical interactions ensure that collagen hydrogels achieve the necessary structural, mechanical, and optical properties to serve as effective corneal subsites, thereby holding significant potential for biomedical applications. However, electric field-assisted fabrication (e.g., electrospinning, electrophoresis) often involves complex setups, specialized equipment, and high costs, hindering large-scale production for clinical use.

### Crosslinking

Although fibrillar collagen structures can be improved physically as an artificial corneal implant, chemical approaches are frequently preferred due to their available functional groups, inherent simplicity, enhanced user-friendliness, and greater ease of application in scientific contexts. This process requires a chemical agent that can induce stable intra- or intermolecular chemical bond formation. Chemical agents that can be used in this process should enable the interaction of collagen through functional groups (amino- and carboxyl-) to lead to the formation of crosslinks between collagen molecules^65^. The bottleneck of the collagen-based artificial corneas lies in the crosslinking method. These crosslinking chemical agents can be toxic and biologically incompatible that usually reduces their translatable application. For instance, the low biocompatibility greatly limits further application of crosslinked collagen in artificial cornea when crosslinked with glutaraldehyde and hexamethylene diisocyanate, as they exhibited potential cytotoxicity^66–70^. Alternatively, a low cytotoxic crosslinker such as carbodiimide may result in low mechanical strength^71–74^. In this section, we will systematically discuss the potential of collagen chemical crosslinking approaches and their utility in enabling the use of artificial corneas.

### Ultraviolet light crosslinking and dehydrothermal treatment

**Fig. 3:**
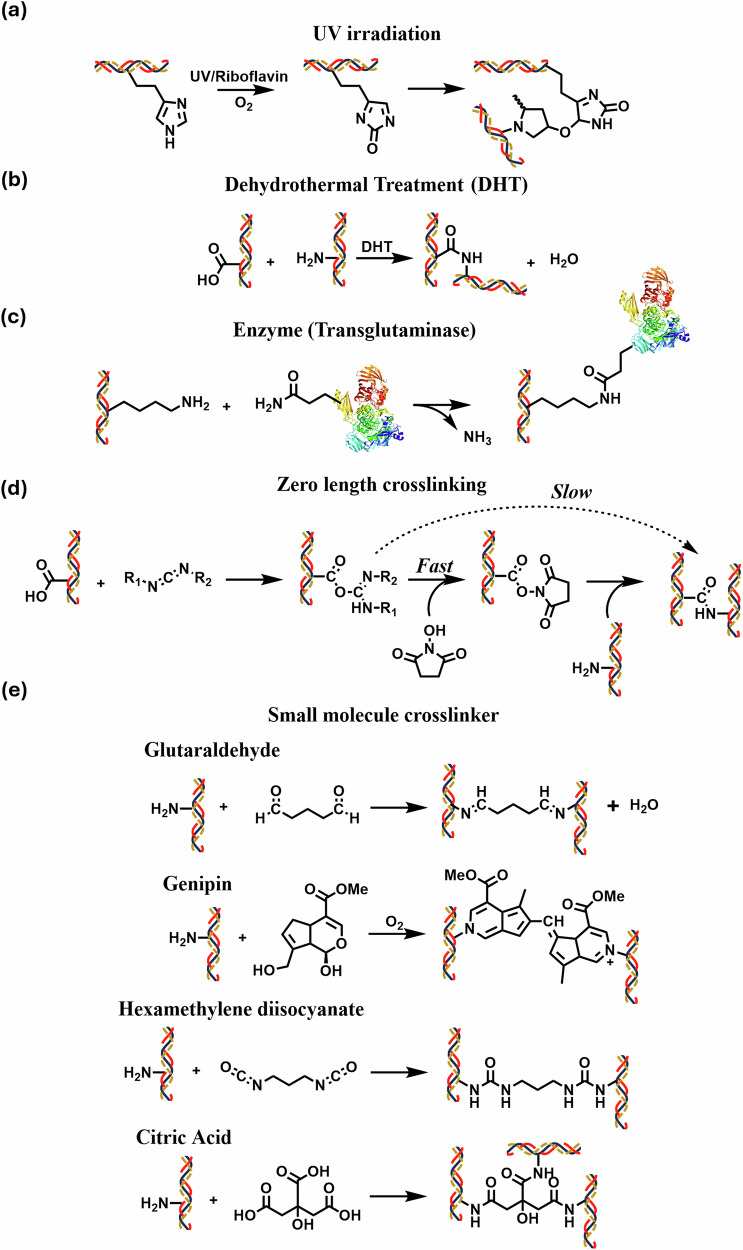
Collagen Crosslinking Strategies. Chemical structures of **a** Ultraviolet light collagen crosslinking, **b** Dehydrothermal treatment collagen crosslinking, **c** Transglutaminase enzymatic crosslinking, **d** Zero-length crosslinking of collagen via EDC/NHS, and **e** Small-molecule crosslinkers for collagen^25^.

Ultraviolet crosslinking and dehydrothermal treatment are external physical factors that can induce chemical crosslinks between collagen fibers. Ultraviolet crosslinking produces free radicals on tyrosine and phenylalanine to crosslink collagen (Fig. 3a). When combining Ultraviolet-A rays at 370 nm with riboflavin, the crosslinking efficiency is significantly enhanced^75^. Riboflavin serves as a photosensitizer that generates oxygen-free radicals to crosslink collagen, while also providing a “shielding effect” by absorbing 90% of UV-A radiation to protect deeper ocular structures. Hence, guaranteeing safe and effective crosslinking of corneal collagen to prevent progression of keratoconus and improve quality of life^76^. However, due to the non-selective and energy-intensive nature, UV-irradiated crosslinking is less suited for biological systems. In addition, dehydrothermal treatment for collagen crosslinking can be achieved by exposing collagen to elevated temperature (>90 °C) under vacuum conditions (Fig. 3b). In this process, water molecules that escape from collagen lead to the formation of intramolecular amide bonds between collagen’s carboxylic acid and amino side groups. These bonds are subsequently crosslinked through condensation in a vacuum oven^77^. With extended reaction time, additional crosslinks can form due to the interactions between lysine and alanine. Although dehydrothermal treatment is slow, it has also been utilized to construct collagen-based corneal stroma, benefiting from its non-cytotoxic process that improves collagen mechanical strength while providing sterilization^78^. However, elevated temperature induces collagen protein structure denaturation, which may be a symptom of its instability under physical conditions that significantly hinders their further usage. On the other side, denatured collagen matrices demonstrate enhanced biocompatibility over native counterparts, retaining potential as functional biomaterials^78,79^.

### Enzymatic crosslinking: transglutaminase

Under physiological conditions, collagen undergoes a series of complex post-translational modifications to maintain its structure and ensure its role in preserving connective tissue integrity. One key modification is the action of lysyl oxidase, an enzyme that converts lysine and hydroxylysine residues into aldehyde forms, such as allysine and hydroxy-allysine. These aldehydes react with neighboring amino groups, forming immature crosslinks, which later develop into stable, covalent crosslinks, strengthening collagen fibrils^80,81^. Enzymatic crosslinking can also create bonds that are compatible with cells and are naturally present in vivo. Transglutaminases are calcium-dependent enzymes that catalyze the post-translational modification of proteins through an acyl transfer reaction between g-carboxamide group and amines, forming ε-(γ-glutamyl)-lysine covalent bonds, which are highly resistant to proteolysis (Fig. 3c)^82^. Transglutaminase-based crosslinking produces collagen hydrogels with enhanced resistance to mechanical stress and proteolytic degradation, excelling in applications requiring specificity, biocompatibility, and gentle processing while being less toxic to cells than chemical crosslinkers^83,84^.

### Chemically induced crosslinking

Covalent crosslinking of collagen involves the connection or bonding of amino acid residues in collagen chains through covalent bonds. This method is commonly used to enhance the mechanical properties and stability of collagen products for use in artificial corneas. Chemical crosslinkers are broadly classified by their potential to integrate directly into the protein. Zero-length crosslinkers modify the local chemical bond of collagen without adding extra chemical moieties to the system, while non-zero-length crosslinkers incorporate a moiety of the crosslinkers into the collagen chains. Nonetheless, there are concerns regarding the potential release of cytotoxic products from non-zero-length crosslinkers when metabolized. Therefore, the choice of crosslinking agents is critical for optimizing the performance and biocompatibility of collagen-based materials in corneal applications.

### Zero-length crosslinking

The use of carbodiimides is one of the most popular and versatile methods for labeling or crosslinking to carboxylic acids. This approach is particularly attractive in collagen crosslinking, as it provides a safe and effective method for stabilizing collagen in applications such as artificial corneal implants^85^. By reacting with carboxylic acids, carbodiimides create reactive intermediates that then interact with amine groups on adjacent polypeptide chains, forming peptide-like covalent bonds (Fig. 3d). The combination of carbodiimides with *N*-hydroxysuccinimide (NHS) enhances crosslinking efficiency while maintaining zero-length crosslinking, as NHS does not incorporate into the final amide bonds. Although this reaction's by-products (urea derivatives) exhibit a certain degree of cytotoxicity, they are soluble in water and can be washed out of the hydrogel after crosslinking^86,87^. This approach eliminates the release of toxic degradation products into surrounding tissues^88^. The residues of the crosslinking agent may have a negative effect in case of insufficient washing after processing the product. Additionally, the hydrogel elasticity of crosslinked collagen is inversely proportional to the concentration of crosslinking agent; hydrogels with a high concentration of EDC are found to be less elastic. Therefore, the concentration of crosslinking agents still requires further optimization to fabricate implants with mechanical properties that match those of the cornea^89^. The most commonly used carbodiimide is water-soluble 1-Ethyl-3-(3-dimethylaminopropyl)carbodiimide (EDC), which usually works with NHS to improve the reaction efficiency. *N*-Cyclohexyl-*N’*-(2-morpholinoethyl) carbodiimide metho-*p*-toluenesulfonate was evaluated as a substitute carbodiimide for corneal applications based on its heterocyclic composition, which reduces molecular mobility and enhances steric hindrance compared to conventional agents. The resultant crosslinked collagen had superior tensile strength and maintained high stability during collagenase degradation^90,91^. EDC/NHS crosslinking can be applied to collagen hydrogels either by premixing with the collagen solution before casting or post-fabrication via immersion, and the formation of covalent crosslinking between collagen fibrils reduces the hydrogel hydrophilicity and wettability^92^.

### Non-zero crosslinking

Non-zero collagen crosslinkers encompass both small-molecule and polymer crosslinkers. Glycation is a non-enzymatic glycosylation, which refers to the covalent attachment of a sugar to a protein, lipid, or nucleic acid molecule^93^. Under hyperglycaemic conditions, the crosslinking of collagen by glycation naturally occurs in the body. In the presence of glucose or other non-toxic reducing sugars, irreversible crosslinking occurs between aldehyde groups on the reducing sugar and amino groups on collagen. The modification of a collagen hydrogel can be achieved by the variation of reducing sugar content. A significant drawback of glycation crosslinking is its tendency to induce dysfunction in the extracellular matrix by disrupting the interactions between collagen, cells, and other extracellular matrix proteins^94–97^. Besides, collagen amine groups react with glutaraldehyde at neutral pH through a Schiff base intermediate to yield an imine bond, for fast and efficient crosslinking that can enhance crosslinked collagen mechanical properties and increase resistance to degradation. Glutaraldehyde forms a tightly crosslinked network and significantly enhances the tensile strength and durability of the collagen scaffold while reducing its antigenicity. However, glutaraldehyde causes significant cytotoxicity and inflammation, thus drastically reducing the biocompatibility of crosslinked collagen. A detoxification strategy has been developed to wash away free aldehyde using glycine or citric acid solutions^98^. Epoxy-based crosslinker, 1,4-Butanediol diglycidyl ether, was also investigated in the fabrication of collagen-based corneal substitutes and has been demonstrated to improve the elasticity and tensile strength of the collagen implants. This crosslinking system exhibited high tailorability of the mechanical properties depending on the coupling time, pH, and concentration of crosslinkers^99^.

Genipin is a natural crosslinking agent that is extracted from Genipa americana fruit. It contains hydroxyls that can spontaneously react with amino acids and crosslink collagen monomers by the formation of Schiff base (Fig. 3e). The structure and properties of the formed hydrogels show a direct relationship to the Genipin concentration^100^. Genipin is biodegradable and has low cytotoxicity, but a high cost of manufacture limits its usage to laboratory-based experimental studies^101^. Additionally, the blue residues that generate during the crosslinking process reduced its transparency and thus affected light penetration through the crosslinked material^102^. Cyanoacrylate glue can be used to temporarily seal the cornea to maintain globe integrity. However, cyanoacrylate has poor biocompatibility and can cause inflammation. Further, incomplete polymerization of cyanoacrylate leaves behind monomers that can undergo hydrolysis to release potentially toxic compounds such as formaldehyde and alkyl cyanoacrylate^103^. Cyanoacrylate is also unpredictable in its duration of adhesion and is generally considered a temporizing repair, with the majority of patients requiring a further intervention. As a biocompatible alternative, citric acid offers a safer crosslinking option. With its three carboxyl groups, citric acid can undergo nucleophilic acyl substitution with the ε-amines of lysine in collagen to form stable amide bonds^25,104^. This reaction, achievable under mild conditions, can enhance the mechanical properties and stability of crosslinked collagen while maintaining cytocompatibility^74^.

**Fig. 4:**
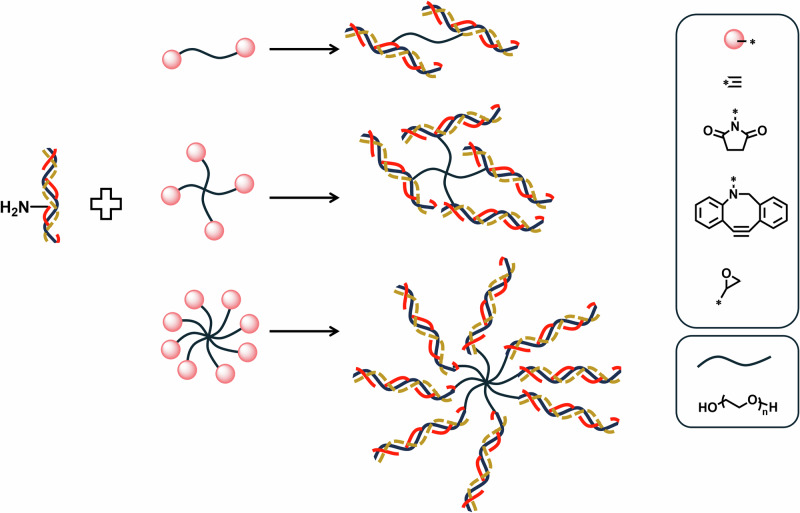
Functional PEG polymer crosslinker for collagen.

In addition, biopolymers with functional groups, such as alkyne, epoxide, *N*-Hydroxysuccinimide, dibenzocyclooctyne, etc., are widely tested for collagen covalent crosslinking. Poly(ethylene glycol) (PEG) is one of the most commonly utilized synthetic polymers used to construct crosslinked collagen hydrogels. Functionalized PEG and its derivatives are frequently combined with collagen to construct scaffold materials and are extensively researched on collagen modification in artificial cornea applications^105,106^. Four-arm PEGs are used as crosslinkers for in situ forming of hydrogel with collagen to repair cornea defects. The formed PEG/collagen matrices are transparent, biocompatible, comfortable for patients, and show bio-integration with native tissue. This type of armed-PEG is typically modified with NHS to form rapid covalent bonding with amine groups from collagen in ambient environments (Fig. 4), which can support multi-layered re-epithelialization following corneal stromal defects^107^. To vary the mechanical properties of collagen hydrogels, 8-arm-PEG has also been used in collagen networks. The higher number of arms increases the modulus at higher PEG concentration but sacrifices the transparency of the hydrogel^108^. Moreover, armed-PEG crosslinked collagen hydrogel can enhance the suture tension of the hydrogel via a denser structure and higher crosslinking degree. Li et al. used alkynyl-modified linear and armed-PEG to form crosslinking between collagen fiber bundles through amino-yne click reaction^109^. Collagen crosslinked with 4-arm-PEG diacrylate formed a denser, more dehydration-resistant network than that with linear PEG, leading to improved mechanical performance, most notably in suture strength. Collagen crosslinked with 4-arm-PEG diacrylate formed a denser, more dehydration-resistant network than that with linear PEG, leading to improved mechanical performance, most notably in suture strength. The amino-yne click reaction under mild conditions preserves the collagen’s triple-helix structure, making the 4-arm-PEG crosslinked collagen membrane promising for corneal repair. Additionally, di/multi-acrylate PEG crosslinked collagen showed improved hydrolytic stability and resistance to enzymatic degradation, although the requirement for ultraviolet light during in situ gelation poses a limitation for its widespread application^110^. In an effort to combine collagen fibril arrangements and chemical crosslinking in the collagen hydrogel to synergistically promote its mechanical and optical properties. Lei et al. have designed a polyrotaxane multiple aldehyde crosslinker based on the host-guest supramolecules of α-cyclodextrin and PEG for corneal repair^111^. Compared with EDC crosslinked collagen, the polyrotaxane multiple aldehydes PEG crosslinked collagen can bear tight suturing and is prone to remodeling of epithelium and stroma of cornea, attributing to the lamellar structure and chemically bonded networks.

### Interpenetrating collagen hydrogel

**Fig. 5:**
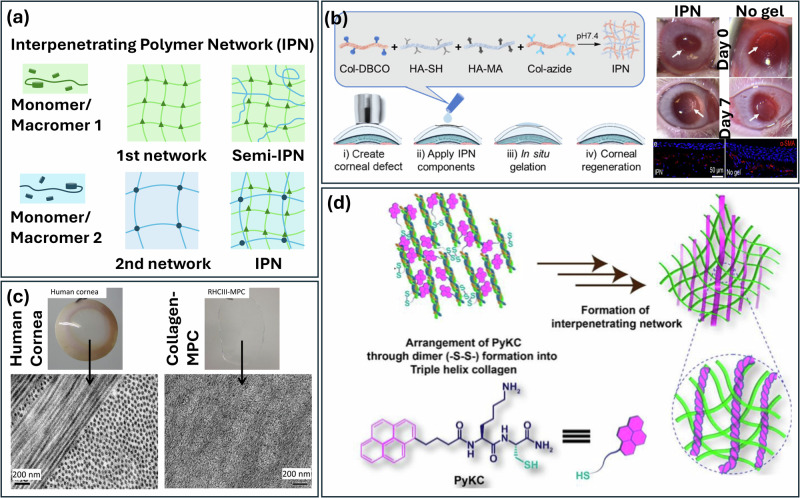
Interpenetrating polymer network based artificial cornea. **a** Synthesis of (Semi)-interpenetrating polymer network (IPN and semi-IPN) hydrogels^115^, **b** Simultaneous interpenetrating polymer network of collagen and hyaluronic acid, photographs before and after IPN treatment and corresponding immunofluorescence staining of regenerated anterior cornea tissue^118^, **c** Optical and SEM images of collagen -MPC INP hydrogel in comparison with native cornea^121^, and **d** Crosslinker-free semi-interpenetrating network^122^.

Other than crosslinking techniques, composite hydrogels are often used to enhance the strength of collagen-based artificial corneas^112^. Interpenetrating polymer network (IPN) hydrogels are a particular category of composite materials designed to enhance the mechanical strength of single-network hydrogels. They are strong candidates to substitute traditional hydrogel for biomedical applications owing to their excellent mechanical properties and their ability to fully replicate aspects of the cellular environment. In an IPN system, two or more networks are interlaced on a polymer scale, the networks can be envisioned to be entangled in such a way that they are concatenated but not covalently bonded to each other and cannot be pulled apart (Fig. 5a). The different polymer networks are interpenetrated but independently crosslinked which exhibit much enhanced mechanical properties and high resistance to wear despite their high-water content. In this regard, the IPN hydrogels of good biocompatibility are considered attractive materials for cornea replacement^113–115^.

In existing studies quantifying the mechanical properties of bioengineered stroma, Young’s modulus and ultimate tensile strength are both critical mechanical characteristics influencing corneal strength, elasticity, cell behavior, and suture retention^116^. The mechanical property of native cornea varies widely across studies, with significant differences attributed to factors such as the region of the stroma analyzed, donor age, storage period, and measurement method. However, the range of Young’s modulus for the human cornea is most consistently reported as 3–13 MPa, while the average tensile strength is 3.81 ± 0.40 MPa. Additionally, as an intraocular graft, a suture is needed during the operation. In order to ensure that the material can be stably stitched within the eye, the suture strength of the materials should be evaluated. It is reported that the suture retention strength of approximately 0.1 N for human amniotic membrane (AM) is a benchmark sufficient to ensure mechanical stability in sutured corneal implants^117^. Consequently, hydrogels intended for corneal implantation should exhibit suture retention forces comparable to or exceeding this threshold (≥0.1 N) to meet clinical requirements. These criteria align with the biomechanical properties validated for human AM in ocular regenerative applications.

Chen et al. invented a simultaneous IPN composed of a collagen network crosslinked by strain-promoted azide-alkyne cycloaddition reaction and a hyaluronic acid (HA) network, which crosslinked via thiol-ene Michael click reaction (Fig. 5b)^118^. Since the crosslinked network is formed in situ in a one-pot system, the IPN hydrogel can be used as a defect filler or self-standing artificial cornea. The IPN gel had a high-water content of 97.35% and maintained high and stable transparency with a refractive index of 1.341. The refractive index was linearly dependent on the concentration of hyaluronic acid; when the HA content reached 45.25%, the refractive index approached that of human cornea. The HA network may also improve the mechanical strength, while the collagen network conferred cell attachment. Methacrylated-gelatin (GelMA), as translucent collagen derivatives, are commonly used for modifying collagen to enhance the mechanical properties without sacrificing the network transparency. GelMA forms one network through photo-initiated free radical polymerization, while the inter-bonding of the collagen layer was achieved by EDC/NHS. The interpenetrating network of GelMA and collagen can provide controllable mechanical properties, and the GelMA backbone contains a large amount of arginine-glycine-aspartic acid (RGD), which can promote cell adhesion^119^. The IPN promotes epithelial overgrowth, tight junction formation in the epithelium, and decreases myofibroblast activity in the injured stroma. Liu et al.^120^ fabricated a bio-interactive corneal substitute by integrating collagen with the phospholipid-mimetic polymer 2-methacryloxyethyl phosphorylcholine (MPC) through a dual-network strategy. The base scaffold consisted of an EDC/NHS-crosslinked porcine atelocollagen network, which delivered structural support and bioactivity. To enhance its biomimetic properties, this network was reinforced with a secondary, interpenetrating component: a poly(ethylene glycol) diacrylate (PEGDA)-crosslinked MPC network. The MPC monomer is a phosphobetaine that replicates the phospholipid composition of cell membranes. The internal structure of the interpenetrated hydrogel, as revealed by TEM, consisted of loosely bundled, fine collagen filaments with a strong alignment (Fig. 5c)^121^. This microstructure, characterized by narrow filament diameters and a high-water content, conferred high transparency to the hydrogel. In addition, the hybrid design leveraged covalent and physical crosslinking to synergize the properties of both components: collagen ensured cell-friendly topography and mechanical resilience, while MPC contributed hydrophilicity, anti-fouling behavior, and enzymatic resistance. Crucially, MPC’s phospholipid-mimicking structure reduced non-specific protein adsorption and enhanced hydrogel stability against collagenase and UV degradation, while the dual-network architecture distributed mechanical stress to resist deformation. Additionally, hydrogels retained the full bioactive, cell-friendly properties of collagen in promoting corneal cell and nerve ingrowth and regeneration. The IPN was designed to leverage the complementary properties of both macromolecules, balancing physical properties with biocompatibility. This hydrogel enables interaction with host tissues to promote tissue regeneration by mobilizing endogenous host stem or progenitor cells, making it a potential candidate for future corneal matrix substitutes.

Semi-IPNs are also formed by the incorporation of a secondary polymer within a crosslinked single-network hydrogel. Similar to IPN, semi-IPN hydrogels can be formed by infusing a second polymer into a crosslinked primary network or selectively crosslinking the primary layer in the presence of the second polymer (Fig. 5a). However, in semi-INPs, the secondary network can be separated from the primary network without breaking any chemical bonds. Hence, semi-IPN has lower mechanical properties but higher flexibility. Although semi-INPs showed inferior mechanical properties to IPNs, they are also chemically more robust than single-network hydrogels and have gained additional attention for use in sustained drug delivery and their ability to regulate cell behaviors^115^. To fabricate a semi-IPN for implantable artificial corneas, Ding et al. synthesized a collagen-polyacrylamide semi-interpenetrating network by polymerizing acrylamide in situ in collagen solution. The formed semi-interpenetrating network gained toughness and mechanical properties of composites. In addition, the organic polymer prepared by in situ free radical polymerization generated relatively uniform pore structures. However, chemical crosslinking modified the parent functional groups of collagens, altering the chemical identity and functional group content. Islam et al. synthesized corneal collagen implants that use crosslinker-free supramolecular gelation strategy by intertwining collagen molecules inside of pyrene conjugated dipeptide amphiphile network (Fig. 5d). This crosslinker-free collagen-based corneal implants had enhanced suturing ability and resistance to enzymatic degradation compared to standard collagen implants without compromising their biocompatibility^122^.

## Functionalization

### Mechanical robust

Crosslinking and constructing IPN within conventional collagen hydrogels can improve their inferior mechanical properties to facilitate clinical translation. However, the inability to be sutured to host tissue and to be sustained with the intraocular pressure pose challenges for their translation. When a suture site is applied to a specific area of the natural tissue, the tissue initially absorbs and disperses the stress at a macromolecular level, and when the localized stress exceeds a certain threshold, the force is transmitted to a more extensive system and distributed across a broader area. Hydrogel based implant that lack hierarchical architecture to effectively dissipate exerted stress render them susceptible to fragility at suture points, thereby hindering their viability for transplantation through suturing^123^. Collagen could hardly bear interrupted stitching during routine surgery; on the other hand, the incorporation of strong synthetic polymers could enhance the mechanical strength and ability to suture the material but significantly reduce the optical transmittance. To obtain high mechanical strength of artificial corneas without sacrificing their inherent transparency, a structural design was invented by Sun and coauthors^124^. The artificial cornea consisted of a transparent collagen core connected with a collagen poly(*Ɛ*-caprolactone) rim. The poly(*Ɛ*-caprolactone) contained fix part that was mechanically robust and convenient for suturing during operation. Although the clinical significance of such a rim-core strategy is yet to be established.

Nanofillers enhance material reinforcement by reducing polymer matrix mobility, increasing entanglements, and altering local structures. In soft tissues, they affect stiffness or softness under mechanical stress. In addition, nanofillers have been shown to increase the elastic and mechanical properties of a hydrogel^125^. The concept of using nanocellulose as reinforcement originated from the possibility of exploiting the high stiffness and strength of cellulose crystals in composite applications^126^. Cellulose nanocrystals, known for their excellent mechanical properties, biodegradability, renewability, adaptable surface chemistry, and optical transparency, are easily modified with chemical methods. This approach has been attempted to enhance the mechanical strength of collagen-based materials for corneal tissue engineering. The mechanical properties were reinforced as the concentration of cellulose nanocrystals increased, while maintaining good swelling performance, optical clarity, and biodegradability, which could meet the requirements for corneal repair^127^. Fiber-reinforced corneal scaffold mimics native tissues’ extracellular matrix structures to guarantee robust mechanical properties, especially suturability. Xu et al.^128^ fabricated a 3D composite material via infusing poly(*Ɛ*-caprolactone) microfibrous scaffold with rat tail type I collagen. The poly(*Ɛ*-caprolactone) scaffolds provide mechanical support, and the orthogonally arranged lamellar structure mimics the native corneal stroma and has the capability to inhibit the differentiation of keratocytes and prevent fibrosis. To further simulate the orthogonal structure of the corneal stroma, the aligned collagen fibrils were stacked into a 3D stromal-mimetic collagen model under an orthogonal arrangement. The introduced topographical cues could support the corneal keratocyte phenotypes in such a way that they hold significant promise as corneal stromal substitutes^129^.

### Cell regulation

**Fig. 6:**
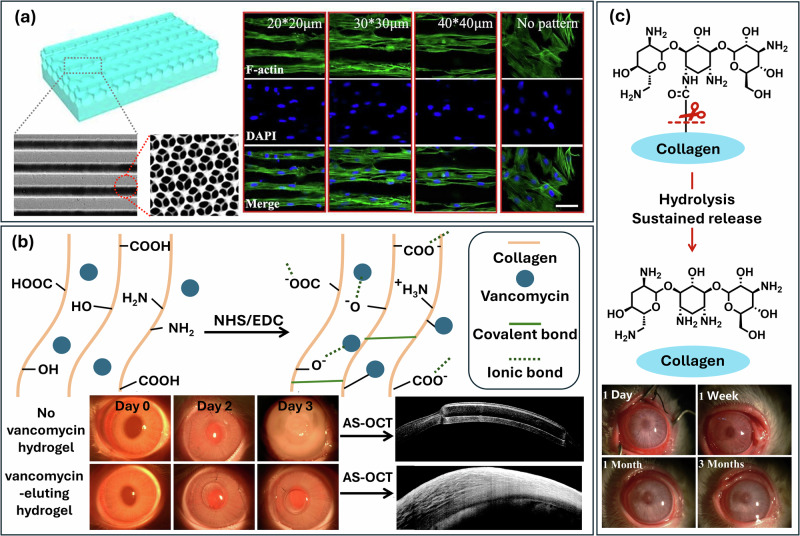
Functional collagen hydrogels for tissue regulation and therapy. **a** Hierarchically structured recombinant human collagen hydrogel guiding cellular alignment^139^, **b** Schematic showing interactions between vancomycin and collagen hydrogel, photography of corneal state before and after surgery and bacteria treatment, and corresponding anterior segment-optical coherence tomography;^143^ **c** The mechanism of tobramycin sustained release process from the Col-Tob film and postoperative observation of LKP using Col-Tob film^141^.

Limbal epithelial stem cells (LESCs) and corneal stromal stem cells (CSSCs) play critical roles in the maintenance and regeneration of the cornea, supporting its structural integrity and transparency throughout life^130^. LESCs are pivotal for corneal surface regeneration, preventing conjunctival ingrowth, neovascularization, and subsequent opacity, which can lead to vision loss. Similarly, CSSCs are essential for the repair of the corneal stroma, ensuring the formation of organized collagen essential for maintaining clarity^131^. In the case of corneal injury, the repair process often leads to disorganized, opaque scar tissue, compromising transparency and potentially causing blindness^132^. In the context of regeneration, innovative techniques such as substrate topographic patterning offer promising tools to influence stem cell behavior and enhance repair outcomes. Patterned substrates can align CSSCs, promoting the production of organized stromal components by manipulating cell shape and orientation. Linear and orthogonal patterns have shown potential to enhance keratocyte-specific gene expression and sulfated glycosaminoglycan synthesis, which are critical for stromal regeneration. These advances illustrate how engineering approaches can influence and complement biological mechanisms to restore corneal transparency and function more effectively^133–135^. Cui et al. developed an efficient method for generating cell-laden, orthogonal-multilayer tissue-engineered corneal stroma, consisting of orthogonally stacked compressed and stretched collagen membranes. This multi-layered scaffold replicated the physiological features of corneal stroma, offering a microenvironment that controls tissue-specific transport and signaling. This is crucial for studying tissue development, regeneration, and disease under conditions that closely mimic the human in vivo context. For example, maintaining the corneal surface topography is critical for cornea reconstruction and regeneration. Collagen films with microgroove surface were demonstrated to greatly affect the orientation, proliferation, migration, and gene expression of corneal epithelial cells and keratocytes^136^. Kilic et al. prepared a ridge-valley patterned collagen scaffold, which aimed to mimic the natural structure and organization of the corneal stroma. Cells in the scaffold were reported to have better attachment and proliferation, and the patterned film also successfully guided the alignment of the cells, and their transparency increased during the culturing. Cornea stroma is characterized by highly organized parallel collagen fibrils, monodisperse in diameter with uniform local interfibrillar spacing^137,138^. To closely mimic this structure, collagen-based hierarchical ordered hydrogel with aligned microgrooves and periodically arranged nanoscale porous structure have been fabricated by combining the technique of lithography and photonic crystal (Fig. 6a). The mechanical properties, transmittance and swelling ratio of these hydrogels, can be adjusted to resemble native cornea tissue. Crytically, in vitro and in vivo studies confirm that they promote aligned cell growth and differentiation, accelerating tissue repair and enabling the regeneration of injured stroma^139^.

### Controlled drug delivery

Although collagen scaffolds have many advantages for replacing pathological corneal tissue, their clinical application is still limited, also due to the risk of microbial infection after implantation, which can lead to serious complications, including loss of vision^140,141^. The primary treatment for managing infectious keratitis (corneal infection) is anti-microbial eye-drop treatment aimed at killing the causal microbe^142^. Current challenges in treating ophthalmic infections include rapid drug loss from the precorneal area and the difficulty in achieving long-term extraocular drug delivery, as well as emerging microbial resistance. Riau et al. developed vancomycin incorporated collage-based artificial cornea scaffolds to address such challenges. In this scaffold, vancomycin is partially bonded with a collagen network through an EDC/NHS crosslinking reaction, leading to delayed drug release due to the crosslinked networks and ionic interactions between vancomycin and collagen (Fig. 6b). On day 3 of post-infection, the implanted corneas without vancomycin elution, were cloudy and edematous with irregular corneal thickness spanning the length of the cornea. Whereas the implanted corneas with vancomycin-eluting hydrogel appeared normal. After transplantation, the localized vancomycin delivery from the collagen hydrogel can prevent implantable device-associated *S. aureus* infection without sacrificing the biomechanical and optical properties of the implant^143^. In order to further enhance the stability of drug-containing hydrogel systems and guarantee a long-term sustained drug release, Liu et al. grafted tobramycin onto collagen hydrogel surfaces. In their study, the release of antibiotics during the first week manifested as a slow and steady release due to hydrolysis between the amino groups of tobramycin and the carboxyl groups of collagens upon the scouring action of the buffer solution (Fig. 6c)^141^. After implantation in the rabbit eye, rejection, neovascularization, and keratoconus were not observed within 3 months.

To prevent rejection and inflammation, anti-inflammatory medications are typically administered after corneal transplantation surgery, which introduces new challenges, such as in achieving optimal drug delivery into the eye, especially in low-resource settings^144,145^. Hydrogels have been proposed not only as scaffolds for corneal regeneration but also as effective vehicles for ocular drug delivery^146–148^. For example, aminoxyl radical oxidized nanocellulose was incorporated with collagen to form a dual crosslinked hydrogel system via EDC/NHS and riboflavin, and dexamethasone has been loaded into the hydrogel, allowing for a sustained release over at least two months to provide sustained anti-inflammatory activity^149^. Our team developed a fully integrative theranostic system with therapeutics and diagnostic capabilities. We explored the potential of a pro-regenerative, collagen-based corneal implant incorporating acyclovir, a widely used antiviral drug, along with gold nanoparticles to create a medicated device capable of regulated drug release following transplantation. Additionally, by replacing the gold nanoparticles with iron nanoparticles, the system was adapted for traceability under magnetic resonance imaging (MRI). This system holds the potential to prevent perioperative reactivation of HSV-1 viruses in compromised corneas. Furthermore, it could be utilized to deliver drugs and other bioactive molecules to the cornea while enabling precise monitoring through a combination of MRI and clinically available in vivo confocal microscopy^150^.

### Collagen functionalization

Suturing remains the prevailing method for securing artificial corneal implants, although with significant drawbacks. The retained sutures may lead to delayed wound healing, early inflammation, and significant early thinning in patient corneas. Therefore, a suture-less strategy is commonly used for clear corneal wounds during cataract surgery^151^. Bio-orthogonal chemistry has been demonstrated to be very useful in developing biomaterials for encapsulating and delivering therapeutic cells, drugs, or cell-derived factors for various medical applications. Rosenquist et al.^152^ used *DL-N*-acetylhomocysteine thiolactone to modify type I collagen, resulting in thiol-functionalized collagen. Through thiol-maleimide click chemistry, the thiol-collagen was then reacted with maleimide-armed PEGs to seal corneal perforations. The introduced bulk thiol groups not only enable rapid gelation but also promote collagen chain self-assembly, enhancing the material’s transparency. The in situ formed collagen-PEG system offers controllable mechanical properties, making it well-suited for suture-less corneal repair, with the added potential to deliver therapeutic cells and factors. Strain-promoted azide-alkyne cycloaddition reaction is another promising bio-orthogonal reaction in the application of suture-less repair of corneal defects. Organic azides and alkynes are relatively biofriendly and less likely to react with cells and tissues, but proceed to a fast cycloaddition reaction when they encounter each other. Collagen modified with azide and dibenzocyclooctyne groups enables the strain-promoted azide-alkyne cycloaddition reaction and induces fast crosslinking between collagen chains. To further mimic the native cornea structure, the authors encapsulated primary corneal keratocytes within crosslinked collagen matrices^153^.

In addition, collagen can also act as an additive material to improve the stability and biocompatibility of synthetic devices. Through bridges of isocyanates, collagen molecules have been immobilized onto a poly(vinyl alcohol) (PVA) scaffold. The collagen-modified PVA scaffold has been reported to be utilized as a keratoprosthesis material that permits surface epithelialization. It reveals good permeability of nutrients and supports a stratified epithelium in vitro. Although the grafts were easy to maneuver, unlike the human cornea, inflammation caused by sutures and looseness induced further detachment of the epithelium. Therefore, despite PVA-col supporting the epithelial growth, adjustment to the surgical procedure will be required to sustain the epithelialized scaffold in vivo^154^.

### Fabrication techniques

Several bioengineering approaches have been attempted to fabricate corneal equivalents based on collagen, by mold casting, 3D printing, electrospinning, and a combination of these processes. Casting is the simplest and cheapest scaffold fabrication technique, which involves creating a mold that can be further filled with biomaterials to form a corneal scaffold. Casting can be less sophisticated when compared to 3D printing and electrospinning, but it is often used in conjunction with these methods to create hybrid scaffolds that benefit from the strength of multiple fabrication techniques^155^. Casted constructs have been demonstrated to have acceptable mechanical properties and optical transmittance and can support corneal cells' adhesion, migration, proliferation, and differentiation. However, they can only provide the scaffold and fail to mimic the natural microenvironment of the native complex corneal tissue, such as the stroma^156^. With the advancement of tissue engineering and various bioprinting techniques, technological innovations such as near-field electrospinning and curvature 3D printing have emerged in customized artificial corneal scaffolds.

### Electrospinning

The microstructure and organization of the stroma are important for its biaxial stiffness and transparency, along with the stroma’s topography, which can influence cell and tissue growth. When cells interact with a surface, their orientation and organization undergo alterations, leading to polarized movements that can alter the strength of their adhesion and activate signaling pathways^157,158^. Electrospun fibers have been used to enhance the mechanical properties of scaffolds for tissue engineering and can also promote cell growth owing to their micro/nanostructures. Due to the high porosity and high aspect ratio, electrospun fibers strongly promote cell adhesion, migration, proliferation, and differentiation, making them an area of significant interest for use in tissue-engineered corneas^159,160^. Direct electrospinning of collagen out of solvents such as fluoroalcohols effectively denatures this biopolymer, partly rejecting its purpose for biomimicking scaffolds emulating the collagen structure and function of the extracellular matrix^161^. To address this issue, the use of soluble collagen or composite materials systems are essentially for the development of nanofibrous collagen-based corneal applications.

**Fig. 7:**
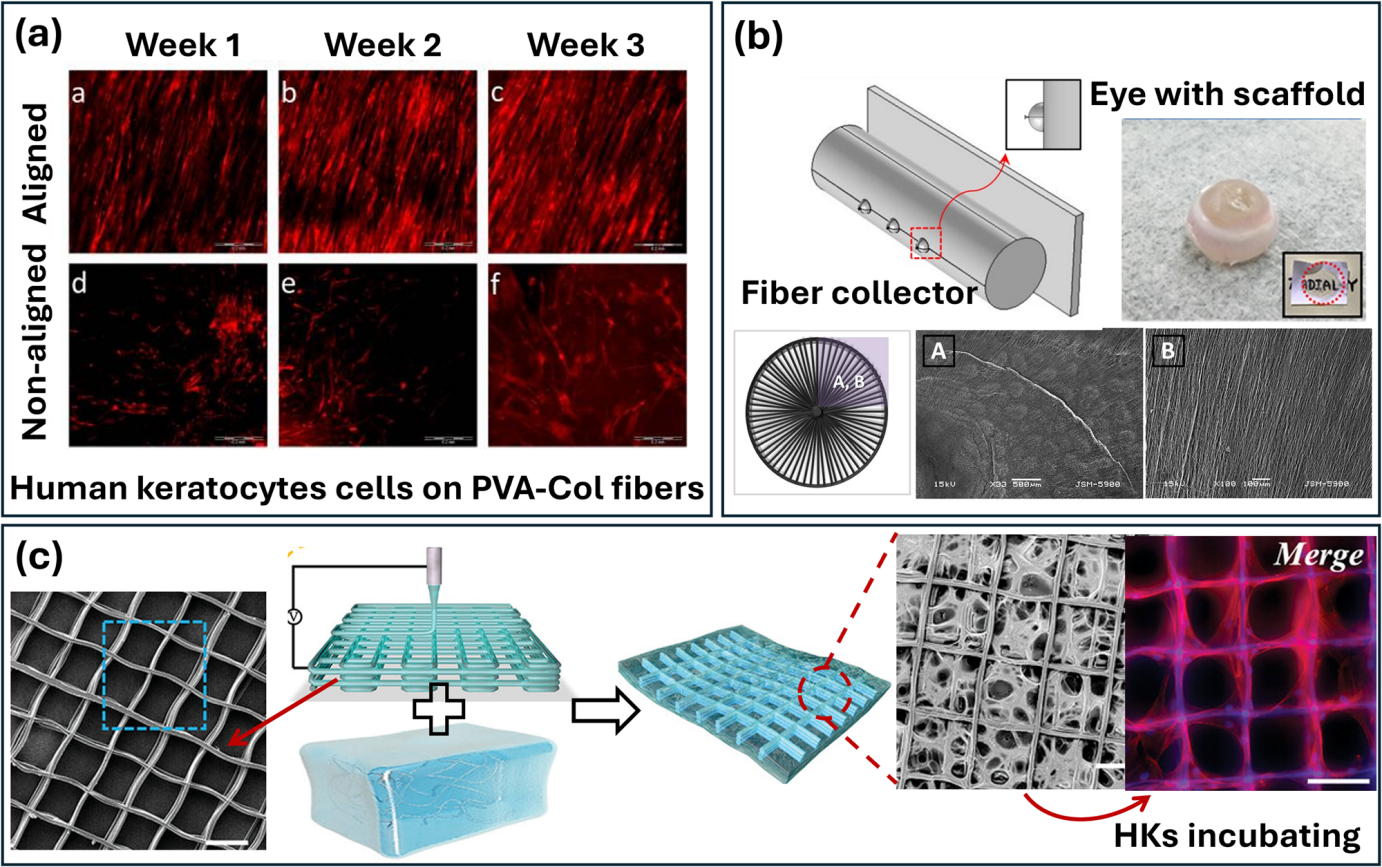
Electrospun collagen scaffolds for corneal tissue engineering. **a** Alignment guidance of PVA-collagen fibers for human keratocytes; **b** Electrospinning fiber collector of the fabrication of 3D radially oriented nanofibrous scaffolds for artificial cornea, and **c** Fabrication and images of near-field electrospinning fiber-reinforced collagen corneal scaffold and human keratocytes incubating^183^.

Nanofibrous scaffolds produced by electrospinning have high surface area to volume ratios and can mimic fibrous structures with interconnecting pores, resembling natural extracellular matrix. They provide support for cell adhesion and movement, proliferation, and differentiation. The properties of electrospun fibers in scaffolds are easy to manipulate, allowing these fibrous scaffolds to provide mechanical support and saturability for implantation^162^. Aligned electrospun fibers can effectively mimic the internal fibril alignment of the cornea stroma, resulting in a transparent and high-strength electrospun corneal scaffold^163^. Electrospun aligned collagen fibers have been used as a matrix for corneal stroma cells. Compared with unaligned fibers, cells grown on aligned collagen fibers had reduced myofibroblast phenotype expression and could downregulate α-smooth muscle actin protein (α-SMA) expression. Electrospun aligned collagen fibers resembling such corneal aligned fibrous platforms have been investigated for engineering corneal replacement tissue^164^. However, the electrospun collagen fibers are not suitable for suturing, which critically limits their clinical application. In order to further enhance the mechanical properties and light transmittance of electrospunned collagen fibers, Wu et al.^165^ spun aligned PVA-collagen composite fibers via electrospinning. By adjusting the composition of PVA and collagen, they obtained a fibrous mat with mechanical strength comparable to that of native corneal tissue. In this study, the aligned structure guaranteed the transmittance, alongside guiding the alignment of cell growth (Fig. 7a). Aligned scaffolds compared to random fiber scaffolds had greater light transmittance, although fall short of the transparency exhibited by the native human cornea. By post-processing an electrospun mat with laser perforation to create holes with different diameters and spacings, the strength and transparency of the mat and of the hybrid construct can be regulated. To achieve this, electrospinning and compressed plastic collagen have been combined to create sandwich-like hybrid constructs with excellent biocompatibility and mechanical properties. Kong et al. optimized laser-perforated macro-scale holes in electrospun poly (lactic-co-glycolide) mats and found that a hole size of 200 μm and a hole spacing of 50 μm provided optimal mechanical properties and light transmittance. In order to mimic the 3D shape of native cornea, Kim et al. developed a 3D electrospinning method to fabricate a hemispherical scaffold to mimic the native properties of the cornea via collagen and poly(*Ɛ*-caprolactone) mixed solution (Fig. 7b). The obtained hemispherical 3D nanofibrous scaffold consisted of radially aligned nanofibers that mimicked the isotropic tissues that grow in a directional orientation. The morphology and topology of the obtained matrix provided a scaffold to be used as a corneal therapeutic platform.

The uniaxial or orthogonal fibers fabricated by electrospinning, however, are only partly aligned due to the limitations of electrospinning in precisely controlling the network architecture. Moreover, the electrospun fibers are very dense, and the cells inoculated on the fiber surface cannot migrate into the inner structure, resulting in a two-dimensional rather than a three-dimensional cell culture. In order to closely simulate the corneal stromal structure, near-field electrospinning, in which there is layer-by-layer assembly of the sub-micro-scale fibers and precise control of the network architecture, has been trialed^156^. Kong et al. developed an ultrasound-responsive adhesive patch for keratitis treatment via near-field electrospinning. The obtained patch was composed of recombinant human collagen hydrogel reinforced with a lattice of square polycaprolactone microfibers (Fig. 7c). For this patch, the precise placement of submicron fibers in an arbitrary layout enabled the emulation of the orthogonal structure of the native corneal stroma. With the incorporation of poly(*Ɛ*-caprolactone) fibers into the collagen patch, the collagen hydrogel exhibited improved mechanical robustness and promoted aligned growth of human keratocytes. The recombinant human collagen hydrogel recreated a 3D microenvironment that emulated the natural structure of the corneal tissue, demonstrating excellent tissue adhesion.

### 3D printing

**Fig. 8:**
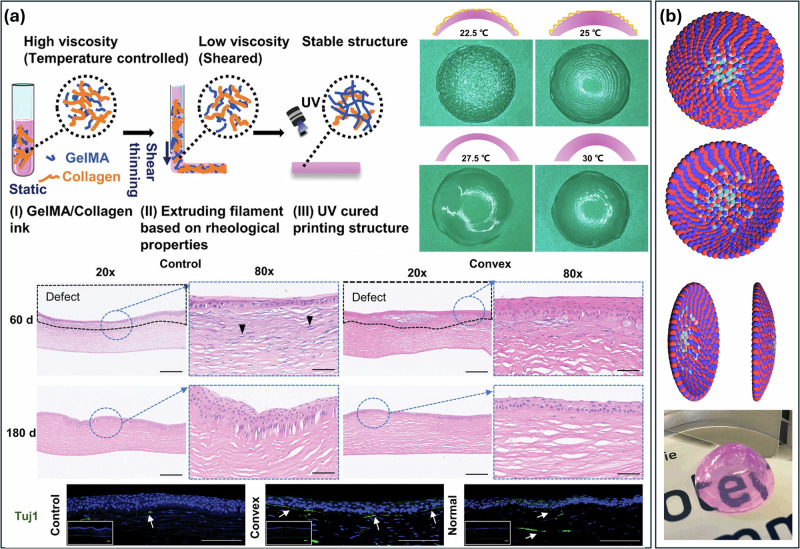
3D bioprinted collagen scaffolds for corneal regeneration. **a** Schematic illustration of printing implants with GelMA/Collagen ink with shear-induced arrangement of fibrils and the images of curvature implants, the Typical hematoxylin and eosin (H&E) staining in the control wound group, convex group, and normal group on days 60 and 180 postsurgery, and the Immunofluorescent staining of corneal neurons (green) in all groups at day 180^182^. **b** 3D model and optical images of drop-on-demand bioprinting scaffold with human corneal stromal keratocytes^184^.

3D printing enables the construction of complex structures without relying on traditional cornea molds. Bioprinting has garnered significant interest for tissue engineering applications due to its capability to direct the hierarchical assembly of three-dimensional biological structures for tissue construction^166^. Unlike conventional tissue engineering, bioprinting supports the precise deposition of bioinks in a prescribed pattern corresponding to the organotypic anatomic cues. 3D bioprinting offers several advantages, including the personalization of refractive power, the ability to create complex multilayer structures, and the introduction of spatial heterogeneities. Techniques for 3D bioprinting encompass pre-printing, modeling, and design. The specific mechanical strength and biocompatibility of the bioinks are critical for the successful biofabrication of a personalized cornea^167^. Kim et al. developed an aligned collagenous structure by inducing shear stress in an atelocollagen extracellular matrix bioink using 3D bioprinting techniques. This artificial cornea demonstrates optimized transparency suitable for cornea transplantation. In this scaffold, the alignment of collagen fibrils occurred along the direction of shear force during extrusion, which was then remodeled along the printing path to create a lattice pattern resembling the structure of the native human cornea. To develop a curved corneal implant to enable the focusing of light, Xu et al. fabricated a convex corneal implant with a smooth surface through 3D printing. GelMA and type I collagen inks with rapid temperature transition property were prepared (Fig. 8a). The resultant implants were curved and could guide cell organization and adhesion; this could promote epithelialization, cell adhesion, and neuron regeneration to heal corneal defects after transplantation. Moreover, bioprinting has emerged as an alternative method for fabricating tissue equivalents using autologous cells, with architectures that resemble the native tissue. This technique employs bioinks that include living cells, growth factors, and extracellular matrix components, allowing for the precise placement of cells within the scaffold. The hematoxylin and eosin (H&E) staining of the control group revealed that a large number of inflammatory cells infiltrated the stroma. There was also a significant decrease in thickness, which is a key indicator for vision. Even on day 180, the control group still had insufficient thickness and showed epithelial hyperplasia. In contrast, in the convex group, it was clear that most of the regenerated and recovered collagen fibers were tight and well-aligned. Most importantly, as illustrated in Fig. 8a, the corneal neuron fibers appeared to be regenerated in the convex group at day 180, indicating that visual functions were recovered. The resulting scaffolds exhibit enhanced structural complexity, biocompatibility, and improved integration^168–170^. Campos et al. proposed a drop-on-demand bioprinting strategy for creating corneal 3D models as suitable implants. Corneal stromal keratocytes were bioprinted in the collagen-based bioinks as 3D biomimetic models. Translucent corneal stromal equivalents with optical properties similar to native corneal stromal tissue were obtained, and the keratocyte cells were viable after the bioprinting process and maintained their native keratocyte phenotypes after in vitro culture (Fig. 8b). Such stromal equivalents could have potential use in the management of patients with corneal stromal diseases and represent a valuable experimental model.

### Preclinical and clinical studies

Penetrating keratoplasty (PK) stands as the most successful and widely accepted treatment for corneal blindness^22^. This procedure involves the full-thickness placement of the damaged tissue with a human donor cornea. In cases where the innermost (posterior) layer of the cornea remains intact, non-penetrating surgical approaches can be employed. These techniques do not penetrate the anterior chamber, significantly reducing the risk of endothelial damage and also avoiding long-term issues such as rejection and decompensation of the endothelial layer. Anterior lamellar keratoplasty (ALK) retains a residual stromal bed, while deep anterior lamellar keratoplasty (DALK) achieves near-total stromal excision down to Descemet membrane^171^. These partial-thickness corneal grafts serve as alternatives to PK, circumventing complications inherent to full-thickness transplantation. By avoiding breaching the anterior chamber, both ALK and DALK eliminate risks of endothelial rejection and graft decompensation. However, their reliance on stromal dissection introduces challenges, including donor-host interface irregularities that may compromise optical clarity and postoperative visual acuity^172,173^. Although innovations in bioengineered corneal substitutes aim to address donor shortages and procedural limitations, allogeneic tissue transplantation remains the dominant clinical paradigm after more than a century of use. This reliance on donor-dependent therapies underscores the urgent need for synthetic alternatives. Collagen, a naturally occurring biopolymer that constitutes the primary component of the corneal ECM, has emerged as a promising biomaterial due to its structural compatibility, tunable properties, and potential to circumvent donor-related constraints^145,174^.

**Fig. 9:**
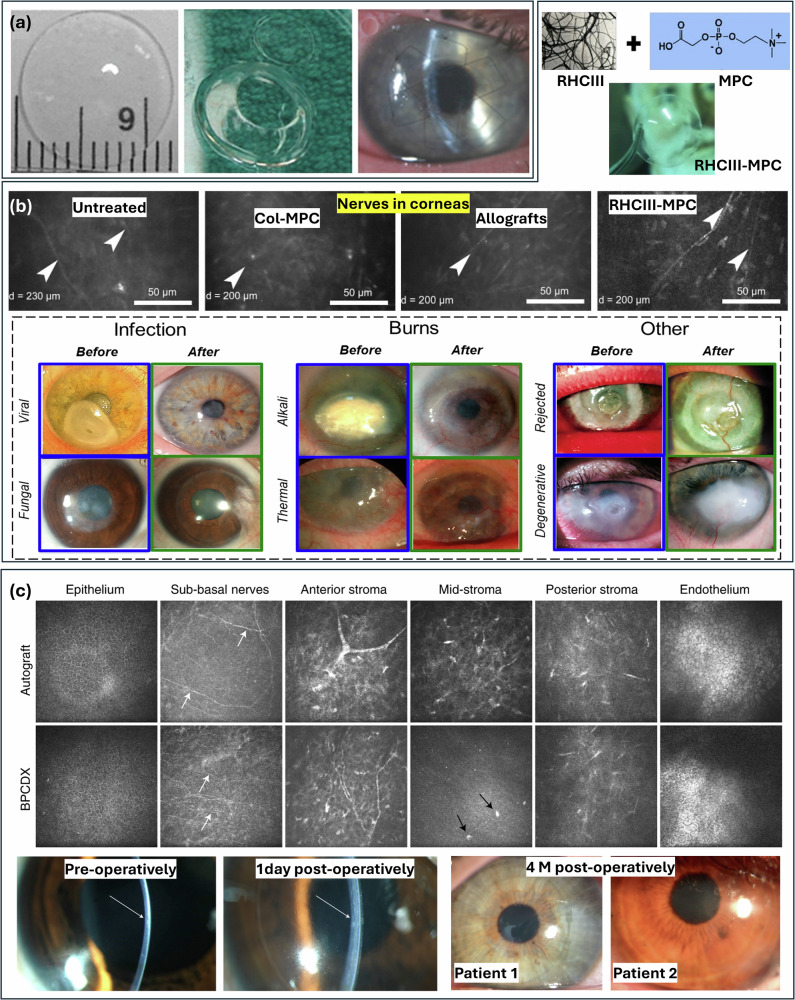
**a** Biosynthetic collagen cornea and ALK implantation method^123^, **b** structure of RHCIII-MPC cornea implants, nerves in cornea of mini-pigs at 12 months postoperative and photographs of patient corneas before and after grafting with RHCIII-MPC implants at 24 months follow-up^185,186^, and **c** in vivo confocal microscopy images of porcine corneas at 6 months and photographs of eyes post BPCDX operative: indicating maintenance of corneal transparency and increase in corneal thickness^187^.

A biosynthetic alternative to human donor tissue, composed of recombinant human collagen, was developed and molded into a artificial cornea. These implants were crosslinked using zero-length crosslinker EDC that avoids incorporation into the hydrated matrix and prevents the release of toxic substances during crosslink breakdown post-implantation. The biosynthetic corneas were optically clear with a refractive index of 1.35 and were trephined to size and implanted by ALK with overlying sutures to secure in place (Fig. 9a)^123^. Fagerholm and colleagues conducted a 24-month follow-up of a Phase 1 clinical study to evaluate the efficacy of these EDC/NHS-crosslinked collagen-based biosynthetic corneal substitutes as an alternative to human donor tissue for corneal regeneration. Ten patients with significant vision loss due to keratoconus or corneal scarring received 500-μm-thick biosynthetic implants. Over the 24-month period, the implants remained stably integrated and avascular, eliminating the need for long-term steroid immunosuppression, which is typically required for traditional allotransplantation. All patients had a morphologically normal regenerated epithelium with good stratification and stable attachment. Nerves regrew into the biosynthetic cornea in 9 out of 10 patients, restoring sensitivity, and the tear film with normal osmolarity was also restored. Without corrective contact lenses, patients with biosynthetic corneas had lower visual acuity than those with donated corneas after 2 years. However, with contact lenses (which they couldn’t wear before surgery), their vision was equivalent. The sutures in the study caused epithelialization issues, but less disruptive ones could solve this problem. With further optimization, these biosynthetic corneal implants could offer a safe and effective solution to the donor cornea shortage. And followed, they provide details of the biosynthetic implants promoted endogenous regeneration of corneal tissue and nerves that were stable over four years, without any rejection episodes and in the absence of immunosuppression. The study suggests that biosynthetic implants, which promote natural regeneration, show promise as long-term solutions for treating corneal blindness^175^.

Corneas with severe pathologies have a high risk of rejection when conventionally grafted with human donor tissues. To address the issue of the shortage of donor tissues and the high risk of rejection associated with allografted corneas, Oleksiy et al. developed bioengineered corneal implants made from interpenetrating networks of crosslinked recombinant human collagen (RHCIII) and 2-methacryloyloxyethyl phosphorylcholine (MPC), which promote tissue integration and prevent neovascularization. The clinical study of RHCIII-MPC implants was carried out with three patients who were considered high-risk patients for limbal epithelial graft rejection, as well as penetrating or lamellar cornea graft rejection and failure. Over the 9–12 months post-operation follow-up period, all three implants remained free of neovascularization or epithelial erosions. Post-operatively, the collagen-MPC corneal implants were fully repopulated with corneal epithelial and stromal cells. Corneal nerves had also re-grown into the implants, as visualized by in vivo confocal microscopy in Fig. 9b^120^. In essence, the implants showed good biocompatibility and stability, improved vision in two patients, and provided surface stability in the third, despite a complication related to pre-existing stem cell deficiency. Three years later, in 2018, M. Mirazul Islam and colleagues significantly expanded the evidence for RHCIII-MPC implants. Patients who had an ulcer or severe scarring due to infections, the implants were well-tolerated and stably incorporated (Fig. 9b). Their work demonstrated safety, efficacy, and mechanistic insights in high-risk patients while refining biomaterial design and therapeutic strategies. This research laid the groundwork for future trials targeting global corneal blindness, particularly in resource-limited settings.

However, the recombinant human collagen could be produced only in small quantities, the implants were also mechanically weak, and the required invasive suturing ALK led to a strong wound-healing response and concerns about long-term stability, including observed partial implant thinning/melting. To address these limitations, Mehrdad Rafat and colleagues developed BPCDX, using abundant, medical-grade porcine dermal collagen. To enhance the implant’s mechanical properties, they employed a novel double crosslinking strategy combining EDC/NHS (chemical) and riboflavin (photochemical) treatments. This approach significantly improved implant strength and resistance to degradation. For implantation, a new minimally invasive, suture-free intrastromal surgical technique was utilized to promote corneal thickening, reshaping, and rapid wound healing. Post-operatively, the epithelial cell mosaic remained intact in both the allograft and BPCDX groups. Basal epithelium and sub-basal nerves (white arrows) were also observed, indicating preservation of the nerve plexus owing to minimal trauma during surgery. Clinical results demonstrate that intrastromal BPCDX implantation is a safe procedure. It effectively reverses pathological corneal thinning and steepening in advanced keratoconus. This leads to substantial and stable improvements in visual and functional outcomes, including contact lens tolerance. These outcomes are comparable to, or potentially superior to, standard donor transplantation. Crucially, BPCDX achieves this with a simpler, safer surgical approach and eliminates the need for donor tissue (Fig. 9c). As these collagen-based implants progress through regulatory approvals, they hold promise to transform corneal transplantation into a standardized, off-the-shelf procedure with improved predictability and reduced complications compared to conventional penetrating keratoplasty. Future research should also investigate the potential for these biomaterials to serve as platforms for delivering therapeutic agents to enhance wound healing and nerve regeneration.

### Conclusions and future perspectives

Integrating collagen into artificial corneal implants represents the most promising approach to address corneal blindness and the global shortage of donor corneas for transplantation. As a primary structural protein in the cornea, collagen offers intrinsic properties such as biocompatibility and structural similarity to the native corneal tissue. These characteristics make collagen an ideal candidate for developing artificial corneal implants, positioning collagen-based substitutes as a promising frontier in corneal tissue engineering. Due to collagen’s potential for seamless integration with host tissue in the corneal environment, collagen-based corneas offer a viable solution to some of the most pressing challenges in corneal replacement and regeneration. Through careful manipulation of physical interactions, crosslinking techniques, and the creation of interpenetrating collagen hydrogels, researchers have made significant strides in enhancing the mechanical strength, transparency, and overall functionality of collagen-based constructs. Additionally, functionalization and advanced fabrication techniques have further optimized these materials, enabling them to mimic the complex architecture of the native cornea more closely.

However, despite the advances in collagen-based corneal substitutes, several challenges remain. The mechanical properties of these constructs, although improved, still require further enhancement to fully match the robustness of the native cornea. Moreover, the long-term biocompatibility and integration of these materials within the host tissue remain critical concerns that need to be thoroughly addressed through extended preclinical and clinical studies. The continued development of collagen-based artificial corneas will likely benefit from a multidisciplinary approach that integrates advances in biomaterials, nanotechnology, and tissue engineering. Innovations in crosslinking methods and the incorporation of bioactive molecules could further improve the mechanical and biological performance of these constructs. Additionally, the use of emerging technologies such as 3D bioprinting and biofabrication may allow for more precise replication of the corneal microarchitecture, potentially leading to personalization, better integration, and functionality of the implanted tissues. Preclinical and clinical trials demonstrate artificial collagen corneas hold significant promise as a revolutionary solution in the field of ophthalmology, yet also face several challenges that need to be addressed for widespread adoption. Collaborative efforts between material scientists, biologists, and clinicians will be essential to overcoming the remaining obstacles and translating these innovative solutions into clinical practice. As research continues to evolve, collagen-based artificial corneas hold the promise of becoming a viable and widely accessible alternative to donor corneas, thereby addressing a critical need in the treatment of corneal blindness and related disorders.

## Data Availability

No datasets were generated or analyzed during the current study.
